# Prior intake of new oral anticoagulants adversely affects outcome following surgery for acute type A aortic dissection

**DOI:** 10.1093/icvts/ivac037

**Published:** 2022-03-08

**Authors:** Juri Sromicki, Mathias Van Hemelrijck, Martin O Schmiady, Bernard Krüger, Mohammed Morjan, Dominique Bettex, Paul R Vogt, Thierry P Carrel, Carlos-A Mestres

**Affiliations:** 1 Department of Cardiac Surgery, University Hospital Zurich, Zurich, Switzerland; 2 Institute of Anesthesiology. University Hospital Zurich, Zurich, Switzerland; 3 Department of Cardiac Surgery, Herzzentrum Duisburg, Duisburg, Germany; 4 Institute of Anesthesiology, University Zurich and University Hospital Zurich, Zurich, Switzerland

**Keywords:** Acute aortic syndrome, Aortic dissection, Type A dissection, Oral anticoagulation, Coumadin, DOAC, NOAC, Bleeding

## Abstract

**OBJECTIVES:**

Oral anticoagulation prior to emergency surgery is associated with an increased risk of perioperative bleeding, especially when this therapy cannot be discontinued or reversed in time. The goal of this study was to analyse the impact of different oral anticoagulants on the outcome of patients who underwent emergency surgery for acute type A aortic dissection (ATAAD).

**METHODS:**

This was a single-centre retrospective study of patients treated with oral anticoagulation at the time of surgery for ATAAD. Outcomes of patients on new oral anticoagulant (NOAC) therapy were compared to respective outcomes of patients on Coumadin. Additionally, a survival analysis was performed comparing these 2 groups with patients who were operated on with no prior anticoagulation.

**RESULTS:**

Between January 2013 and April 2020, a total of 437 patients (63.8 ± 11.8 years, 68.4% male) received emergency surgery for ATAAD; 35 (8%) were taking oral anticoagulation at the time of hospital admission: 20 received phenprocoumon; 14, rivaroxaban; and 1, dabigatran. Compared to Coumadin, NOAC was associated with a greater need for blood-product transfusions and haemodynamic compromise. Operative mortality was 53% in the NOAC group and 30% in the Coumadin group. A 5-year survival analysis showed no significant difference between the NOAC and the Coumadin group (*P* = 0.059). Compared to 402 patients treated during the study period without anticoagulation, patients taking NOAC had significantly worse survival (*P* = 0.001), whereas that effect was not observed in patients undergoing surgery who were taking Coumadin (*P* = 0.99).

**CONCLUSIONS:**

Emergency surgery for ATAAD in patients taking NOAC is associated with high morbidity and mortality. NOAC are a major risk factor for uncontrollable bleeding and haemodynamic compromise. New treatment strategies must be defined to improve surgical outcomes in these high-risk patients.

**Figure:**
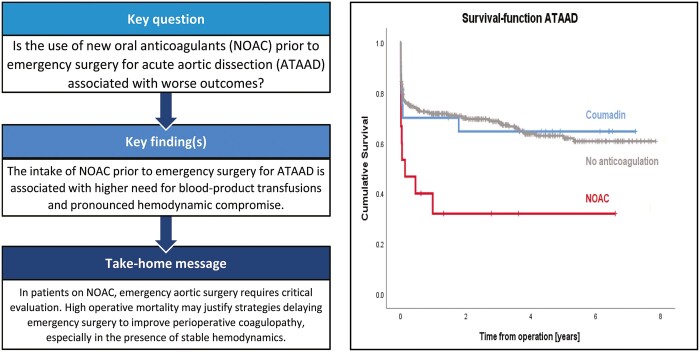

## INTRODUCTION

The increasingly liberal use of new oral anticoagulants (NOAC) represents a challenge when dealing with patients at high risk of bleeding. Compared to warfarin, NOAC seem to be associated with fewer major, fatal or intracranial bleedings but with an increased risk of gastrointestinal haemorrhage when prescribed for stroke prophylaxis in non-valvular atrial fibrillation [1]. Although andexanet alfa and idarucizumab have recently gained market approval to rapidly reverse the effects of NOAC [2, 3], their use still contributes to high morbidity and mortality due to the limited availability of these “antagonists”, their high costs and scanty experience in patients presenting with a surgical emergency. Recommendations on how to proceed in case of elective cardiac surgery with prior oral anticoagulation (OAC) were issued in the 2017 EACTS/EACTA guidelines on patient blood management for adult cardiac surgery. However, little is known about the impact of OAC in patients having emergency heart surgery.

Acute type A aortic dissection (ATAAD) is a life-threatening condition with high mortality. Any type of organ malperfusion increases the risk of adverse outcome. Few reports give guidance on how to proceed in patients on OAC presenting with ATAAD requiring emergency surgery. The goal of this study was to analyse the outcome of emergency surgery for ATAAD in patients under prior therapy with vitamin K antagonists (VKA) or NOAC in relation to patients treated without prior OAC and to review the evidence for their perioperative management.

## METHODS

### Study design and ethics

This is a retrospective analysis from a single centre’s prospective database on patients undergoing emergency surgery for ATAAD. Patients who were on OAC at hospital admission were identified and, depending on the anticoagulants they were taking (NOAC or Coumadin), divided into 2 groups. The main analysis compared in-hospital outcomes (mortality, haemodynamic compromise, need for transfusions) of these 2 groups. In a subanalysis, a Kaplan–Meier survival analysis was performed, comparing both anticoagulated groups to patients who were operated on for ATAAD during the study period without prior anticoagulation therapy. The database was locked as of April 2020 for completion of follow-up. Informed consent was waived because the main database was approved by the institutional review board/ethics committee (IRB/EC-Nr. 2017–00824) for retrospective analyses of outcomes after ATAAD.

### Patient selection

The University Hospital Zurich (Universitätsspital Zürich—USZ) is an academic tertiary referral centre with a catchment area of approximately 3 million people. Data on demographics, surgical indications, operative notes, complications and outcomes were collected. With the main focus on patients taking oral anticoagulants at hospital admission, all patients who underwent emergency surgical treatment for ATAAD during the study period were analysed.

### Definitions

ATAAD was defined according to the 2014 European Society of Cardiology Guidelines on the diagnosis and treatment of aortic diseases. The DeBakey [4] and Stanford [5] classifications were used to define the extent of aortic wall dissection. The Penn classification [6] defined the presence or absence of branch vessel malperfusion, circulatory collapse or both.

OAC was defined as oral intake of an anticoagulant medication up to the time of surgery. The oral anticoagulation regimens included the VKA phenprocoumon (Marcoumar in Switzerland) and the NOAC rivaroxaban and dabigatran (Xarelto/Pradaxa in Switzerland). Patients taking phenprocoumon are referred to as the Coumadin group; patients taking rivaroxaban and dabigatran, as the NOAC group.

The gold standard for the diagnosis of ATAAD was contrast-enhanced computed tomography (CT) and/or echocardiography.

Operative mortality was defined as 30-day or in-hospital death. Interhospital transfer was not considered as discharge.

### Data collection

Pre-, intra- and postoperative data were collected from the USZ electronic medical record system. Follow-up data were collected from outpatient clinic visits or by directly contacting the patients, their relatives or their primary care physicians. All data were collected or double-checked by the main author. Follow-up was obtained in 100% of the cases.

### Primary and secondary end points

Perioperative death, haemodynamic stability and the need for blood-product transfusions were defined as primary end points. As a secondary end point, the mid-term follow-up survival of each anticoagulated group was estimated. The follow-up survival of both anticoagulated groups and of patients who were treated for ATAAD during the study period without prior anticoagulation was defined as outcomes of interest for the study subanalysis.

### Patient assessment and operative strategy

Patients with an established diagnosis of ATAAD were rapidly transferred to the operating room (OR) if ATAAD was diagnosed in the USZ emergency department or were directly admitted to the OR if the diagnosis of ATAAD had been established elsewhere. All patients were preoperatively evaluated by the staff surgeon on call. Decisions regarding indication and priority were based on clinical presentation and emergency imaging.

Surgical access and the technique for aortic dissection repair were left to the discretion of the operating surgeon. Unless the anatomy was not favourable, the right subclavian artery was prepared and cannulated for cardiopulmonary bypass. Once the cannula was in place, a median sternotomy was performed, the pericardium opened and the right atrium cannulated with a two-stage cannula. A left ventricular vent was placed through the right superior pulmonary vein. Antegrade and/or retrograde cardioplegia cannulae were placed at the surgeon’s discretion. Variations of the sequence of the surgical steps and cannulation sites may have occurred depending on the operating surgeon and the patient’s condition.

The extent of aortic dissection repair included aortic valve resuspension, aortic root replacement with or without a valve-sparing procedure, replacement of the ascending aorta or aortic arch replacement with or without the antegrade delivery of a stent graft (frozen elephant trunk). Whenever needed, a graft anastomosis to the aortic arch was performed with the patient under moderate hypothermic circulatory arrest of the lower body (target temperature 28°C) with bilateral antegrade selective cerebral perfusion.

**Figure 1:**
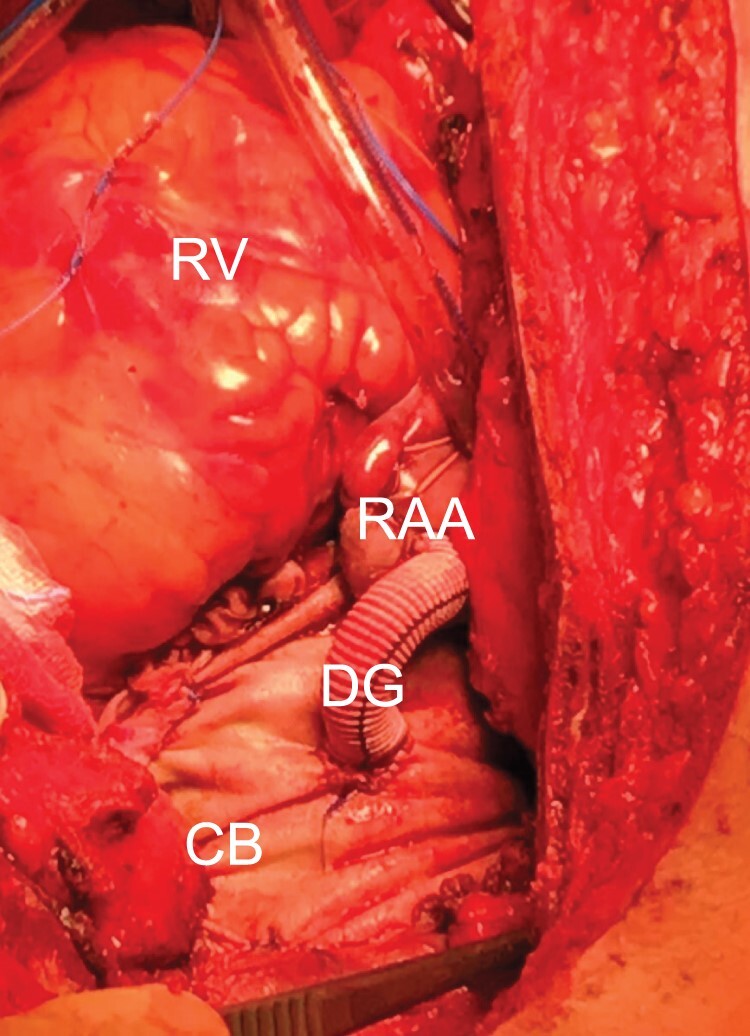
Anaesthetist’s view of a Cabrol fistula from the upper mediastinum into the right atrium after repair of acute type A aortic dissection. CB: Cabrol baffle in the upper mediastinum; DG: 8 -mm Dacron graft; RAA: right atrial appendage; RV: right ventricle.

After weaning the patient off cardiopulmonary bypass, thorough haemostasis was performed. Transfusion of blood products and treatment of coagulopathy were managed by the anaesthesiologist according to institutional guidelines. In addition to conventional laboratory tests, repeated point of care testing, including rotational thromboelastometry was used in every patient to monitor blood coagulation during the operation and in the ICU until bleeding clinically subsided. Treatment of coagulopathy comprised primarily the targeted replenishment of depleted coagulation factors. Fresh frozen plasma was used if coagulation factor V decreased below 20%. Thrombocytes were administered in platelet counts below 80,000/µl. According to the degree of haemodynamic compromise, a blood haemoglobin level higher than 80 g/l was targeted. Surgical maneuvers for severe bleeding included mediastinal packing and the construction of a perigraft to the right atrium or an innominate vein fistula [7] (Fig. 1). In case of prolonged bleeding and/or haemodynamic compromise, the chest was left open.

### Postoperative management and follow-up

Patients were treated by a multidisciplinary team in the intensive care unit (ICU) with outlined principles [8, 9]. For open chest therapy, preemptive antibiotic therapy was continued. Surgical revision, when necessary, was performed in the ICU [10] or in the OR. If the chest remained open, once the coagulation status and bleeding rate were under control, the patient was sent to the OR for delayed sternal closure [11].

## STATISTICAL ANALYSES

Statistical analyses were performed using the SPSS 25.0 software package (IBM Corp. Released 2017. IBM SPSS Statistics for Windows, Version 25.0. Armonk, NY, USA). Standard descriptive statistics were used to summarize data. Continuous variables are presented as means with standard deviation, categorical variables as absolute numbers and proportions. For comparison of categorical data, Fisher’s exact test was used. The Mann-Whitney-Wilcoxon test was used to compare continuous data.

Survival was calculated using the Kaplan–Meier method. The log-rank test with Kaplan–Meier curves was used for group survival comparisons. The estimated survival of a patient started at the time of the operation and ended at the time of death (event) or the latest known follow-up (censored).

A *P*-value < 0.05 was considered statistically significant.

## RESULTS

### Patient characteristics

Between January 2013 and April 2020, a total of 437 consecutive patients (63.8 ± 11.8 years, 68.4% male) received emergency surgery for ATAAD in our institution. Thirty-five patients (8%) were identified with OAC at the time of admission. Fifteen patients were under NOAC (14 rivaroxaban, 1 dabigatran) whereas 20 had received phenprocoumon (Marcoumar). The mean age of the anticoagulated patients was 70.9 ± 9.8 years; 24 (69%) were male. In 63% of cases, atrial fibrillation was the indication for OAC. Except for the Quick/international normalized ratio (INR) at hospital admission, no significant differences in preoperative patient characteristics were found between the NOAC and the Coumadin group (Table 1).

**Table 1:** Preoperative clinical and surgical characteristics of patients with acute type A aortic dissection who were taking new oral anticoagulants

	NOAC group	Coumadin group	*P*-Value
Number of patients [n]	15	20	
Age [years]	69 ± 9	73 ± 11	0.14
Gender			
Male	12 (80%)	12 (60%)	0.28
Female	3 (20%)	8 (40%)	
Body mass index [kg/m^2^]	26.7 ± 4.2	28.6 ± 4.6	0.27
Indication for anticoagulation			
Atrial fibrillation	12 (80%)	10 (50%)	0.089
Pulmonary embolism	2 (13%)	4 (20%)	0.68
Peripheral venous thromboses	1 (7%)	2 (10%)	1.00
Mechanical valve prosthesis	0 (0%)	3 (15%)	0.24
Other	0 (0%)	2 (10%)	0.50
Comorbidities			
Hypertension	13 (87%)	15 (75%)	0.67
Diabetes mellitus	5 (33%)	6 (30%)	1.00
Dyslipidaemia	2 (13%)	1 (5%)	0.57
Ongoing/former smoker	5 (33%)	8 (40%)	0.74
Chronic obstructive pulmonary disease	1 (7%)	6 (30%)	0.20
Chronic renal impairment	3 (20%)	6 (30%)	0.70
History of stroke	3 (20%)	5 (25%)	1.00
Coronary artery disease	3 (20%)	4 (20%)	1.00
Peripheral artery disease	4 (27%)	5 (25%)	1.00
Connective tissue disorder	0 (0%)	0 (0%)	1.00
Previous cardiac surgery	2 (13%)	4 (20%)	0.68
Laboratory findings			
Haemoglobin [g/l]	123 ± 17	117 ± 21	0.52
Haematocrit [%]	37.0 ± 4.6	35.2 ± 5.9	0.42
Platelet count [G/l]	186 ± 88	212 ± 96	0.20
Quick [%]	57 ± 20	28 ± 11	<0.001^*^
INR	1.5 ± 0.3	2.5 ± 0.8	<0.001^*^
aPTT [sek]	50 ± 36	41 ± 32	0.31
Anti-FXa activity (rivaroxaban) [µg/l]	103 ± 148	–	–
Underlying pathology of acute aortic dissection			
Stanford type A	15 (100%)	20 (100%)	1.00
DeBakey type I	9 (60%)	11 (55%)	1.00
DeBakey type II	6 (40%)	9 (45%)	1.00
Penn class a	9 (60%)	14 (70%)	0.72
Penn class b	4 (27%)	2 (10%)	0.37
Penn class c	1 (7%)	2 (10%)	1.00
Penn class b and c	1 (7%)	2 (10%)	1.00
Dissection of supra-aortic vessels	8 (53%)	8 (40%)	0.51
Aortic regurgitation			
None/trivial	3 (20%)	5 (25%)	1.00
Mild	7 (47%)	5 (25%)	0.28
Moderate	3 (20%)	9 (45%)	0.16
Severe	2 (13%)	1 (5%)	0.57
Left ventricular ejection fraction [%]	53 ± 8	53 ± 12	0.63
EuroSCORE II [%]	36.6 ± 17.5	35.1 ± 21.2	0.73
Expected mortality—IRAD model I [%]	24.4 ± 24.3	23.5 ± 22.1	0.83
Expected mortality—IRAD model II [%]	37.6 ± 32.4	23.0 ± 31.3	0.12
GERAADA score [%]	25.2 ± 20.2	18.6 ± 9.3	0.44

aPTT: activated partial thromboplastin time; GERAADA score: German Registry for Acute Aortic Dissection Type A; INR: international normalized ratio; IRAD: International Registry of Acute Aortic Dissection; NOAC: new oral anticoagulants.

### Type of dissection

All 35 patients with prior anticoagulation were diagnosed with ATAAD. Computed tomography scans confirmed the diagnosis in 33 cases, whereas 1 patient who presented primarily with cardiac ischaemia due to occlusion of the left main stem was diagnosed by coronary angiography. In 1 patient with unstable haemodynamics, ATAAD was diagnosed by echocardiography. The dissection was limited to the ascending aorta in 15 (43%) cases (DeBakey type II), 6 (17%) presented with haemodynamic instability due to pericardial tamponade, cardiac ischaemia or severe aortic regurgitation (Penn classification c and b and c) [6].

### Operative strategy and timing

Surgery was delayed in 3 out of the 15 patients under NOAC therapy.

One patient was taken to the OR immediately after diagnosis, but the operation was aborted and delayed due to severely impaired coagulation while preparing the subclavian artery for cardiopulmonary bypass (CPB). In this patient, anti-factor Xa activity (AFXa) for rivaroxaban decreased from 340.8 to 34.3 µg/l within 24 h; the patient was brought back to the OR thereafter.

One patient with ATAAD while taking rivaroxaban and clopidogrel presented with limb ischaemia but was otherwise stable on admission. Immediate fenestration of the infrarenal dissection membrane was performed, followed by external iliac artery stenting. AFXa activity was 147.9 µg/l on admission. Thoracic aortic repair was postponed for a total of 12 h following referral. Full blood haemadsorption (CytoSorb, Cytosorbents, Monmouth Junction, NJ, USA) was performed over an 8 -h period to enhance the blood clearance of rivaroxaban. AFXa activity decreased to 46.1 µg/l, and the patient was then transferred to the OR [12]. Because the coagulation was still impaired, the upper mediastinum in this case was covered with a pericardial patch that drained blood from the periaortic cavity into the right atrium, and the chest was left open for 4 days.

One patient received rivaroxaban because of atrial fibrillation when ATAAD was diagnosed. Because of stable haemodynamics without pericardial effusion and absence of acute chest pain, emergency surgery was delayed for 42 h. AFXa activity spontaneously dropped from 122.3 to 20.0 µg/l without additional treatment.

The other 32 patients were operated on immediately after diagnosis. AFXa activity at the beginning of the operation was 103.2 ± 148.0 µg/l in the NOAC group. The INR and the Quick value were 2.5 ± 0.8 and 28 ± 11% in the Coumadin group vs 1.5 ± 0.3 and 57 ± 20% in the NOAC group (*P* < 0.001). Due to limited availability and experiences, neither andexanet alfa nor idarucizumab was used in these patients.

### Operative data

No significant differences were found with respect to duration of cardiac ischaemia, circulatory arrest or CPB, but patients taking NOAC had longer overall operating times than those taking Coumadin (465 ± 187 min vs 323 ± 73 min, *P* = 0.007). Intraoperative characteristics and surgical procedures are summarized in Table 2. Because of low cardiac output and/or respiratory failure, extracorporeal membrane oxygenation support was established in 4 patients in the NOAC group and in 2 patients in the Coumadin group (*P* = 0.37). Delayed sternal closure occurred significantly more often in the NOAC group (53%) compared to the patients operated on under VKA (15%) (*P* = 0.027). Two patients in the NOAC group required the construction of a Cabrol fistula to prevent excessive postoperative blood loss.

**Table 2:** Intraoperative data for patients with acute type A aortic dissection who were taking new oral anticoagulants

	NOAC group	Coumadin group	*P*-Value
Operative times			
Overall operation [min]	465 ± 187	323 ± 73	0.007^*^
Cardiopulmonary bypass [min]	234 ± 110	188 ± 55	0.24
Cardiac ischaemia [min]	114 ± 37	102 ± 38	0.35
Antegrade cerebral perfusion [min]	62 ± 89	27 ± 16	0.093
Circulatory arrest—lower body [min]	38 ± 25	28 ± 15	0.30
Minimal temperature [°C]	28.2 ± 2.1	28.5 ± 1.9	0.63
Haemadsorption (CytoSorb)	11 (73%)	11 (55%)	0.31
Delayed surgery	3 (20%)	0 (0%)	0.070
Procedures performed			
Root sparing	10 (67%)	12 (60%)	0.74
Root replacement	5 (33%)	8 (40%)	0.74
Ascending replacement	15 (100%)	20 (100%)	1.00
Arch replacement	3 (20%)	1 (5%)	0.29
Debranching of the supra-aortic vessels	3 (20%)	5 (25%)	1.00
Frozen elephant trunk	3 (20%)	0 (0%)	0.070
Additional procedures			
Valve	0 (0%)	1 (5%)	1.00
CABG	2 (13%)	1 (5%)	0.57
Postoperative ECMO	4 (27%)	2 (10%)	0.37
Delayed sternal closure	8 (53%)	3 (15%)	0.027^*^

CABG: coronary artery bypass grafting; ECMO: extracorporeal membrane oxygenation; NOAC: new oral anticoagulants.

### In-hospital outcomes

#### Coumadin versus new oral anticoagulants

Compared to the Coumadin group, patients with prior NOAC required more intraoperative transfusions of packed red blood cells (8.6 ± 8.9 vs 3.4 ± 4.1, *P* = 0.039) and fresh frozen plasma (6.5 ± 6.2 vs 1.1 ± 2.0, *P* = 0.006). Although statistically not significant, the retransfused cell saver volume and the amount of transfused platelet units were higher in the NOAC group (Table 3).

**Table 3:** Outcome data for patients with acute type A aortic dissection who were taking new oral anticoagulants

	NOAC group	Coumadin group	*P*-Value
Exitus in tabula	1 (7%)	1 (5%)	1.00
In-hospital mortality	8 (53%)	6 (30%)	0.19
Cause of death			
Uncontrollable bleeding	5 (63%)	0 (0%)	0.031^*^
Cerebrovascular bleeding/ischaemia	2 (25%)	1 (17%)	1.00
Heart failure	0 (0%)	3 (50%)	0.055
Multiorgan failure	1 (13%)	2 (33%)	0.54
Intraoperative transfusions			
Cell-saver volume [ml]	1977 ± 1733	995 ± 728	0.30
Red blood cells [U]	8.6 ± 8.9	3.4 ± 4.1	0.039^*^
Platelets [U]	3.7 ± 3.5	1.8 ± 1.7	0.064
Fresh frozen plasma [U]	6.5 ± 6.2	1.1 ± 2.0	0.006^*^
Haemodynamics on ICU admission			
Noradrenaline [mcg/min]	23.6 ± 18.4	9.4 ± 8.7	0.004^*^
Adrenaline [mcg/min]	4.3 ± 3.8	1.9 ± 3.6	0.055
Milrinone [mcg/min]	3.5 ± 4.0	1.8 ± 3.4	0.18
Inotropic support	11 (73%)	9 (45%)	0.17
Chest tube output 0-24 h [ml]	1381 ± 1049	1265 ± 982	0.97
Chest tube output 24-48 h [ml]	1039 ± 1066	658 ± 361	0.65
Chest tube output 0-24 h vs 24-48 h	*P* = 0.997	*P* = 0.008^*^	
Transfusions 0-24 h postoperatively			
Red blood cells [U]	6.5 ± 7.4	1.4 ± 2.0	0.20
Platelets [U]	1.9 ± 2.0	0.3 ± 0.6	0.11
Fresh frozen plasma [U]	5.0 ± 5.1	0.5 ± 1.3	0.059
Transfusions 24-48 h postoperatively			
Red blood cells [U]	3.5 ± 7.3	0.9 ± 2.4	0.70
Platelets [U]	0.9 ± 2.2	0.2 ± 0.8	0.53
Fresh frozen plasma [U]	2.5 ± 7.0	0.2 ± 1.0	0.83

ICU: intensive care unit

At the time of transfer to the ICU, 23.6 ± 18.4 mcg/min of noradrenaline were administered to patients in the NOAC group, compared with 9.4 ± 8.7 mcg/min to those in the Coumadin group (*P* = 0.004). Additional inotropic support (adrenaline, milrinone, dobutamine) was required in 11 (73%) patients in the NOAC group and in 9 (45%) in the Coumadin group (*P* = 0.17). Chest tube output during the first 24 h was comparable in both groups (1381 ± 1049ml vs 1265 ± 982ml, *P* = 0.97), but significantly decreased during the following 24 h in the Coumadin group (*P* = 0.008) only.

Operative mortality was 53% in the NOAC group and 30% in the Coumadin group (*P* = 0.19). Cause of death was intractable bleeding in 5 out of 8 deaths in the NOAC group, and heart failure (*n* = 3), multiorgan dysfunction (*n* = 2) and cerebral bleeding (*n* = 1) in the 6 patients in the Coumadin group.

#### Coumadin/new oral anticoagulants versus no anticoagulation

Compared to the operative mortality of 24.4% observed in our centre throughout the study period in patients without prior OAC (Table 4), operative mortality was significantly higher in the NOAC group (*P* = 0.029) but not different in the Coumadin group (*P* = 0.60). The odds ratio for fatal 30-day or in-hospital outcome with prior NOAC was 3.50 [95% confidence interval 1.24–9.90].

**Table 4:** Characteristics of patients with acute type A aortic dissection without prior anticoagulation

	ATAAD without prior OAC
Number of patients [n]	402
Age [years]	64 ± 12
Gender	
Male	274 (68%)
Female	128 (32%)
Body mass index [kg/m^2^]	26.8 ± 5.0
Comorbidities	
Hypertension	273 (68%)
Diabetes mellitus	20 (5%)
Dyslipidaemia	98 (24%)
Ongoing/former smoker	129 (32%)
Chronic obstructive pulmonary disease	41 (10%)
Chronic renal impairment	33 (8%)
History of stroke	15 (4%)
Coronary artery disease	44 (11%)
Peripheral artery disease	21 (5%)
Connective tissue disorder	3 (1%)
Previous cardiac surgery	7 (2%)
Underlying pathology of acute aortic dissection	
Stanford type A	402 (100%)
DeBakey type I	268 (67%)
DeBakey type II	134 (33%)
In-hospital deaths	98 (24.4%)

ATAAD: acute type A aortic dissection; OAC: oral anticoagulants.

### Follow-up outcomes: survival

#### Coumadin versus new oral anticoagulants

One death was reported 2 years after surgery in the Coumadin group. This patient died of metastatic cholangiocarcinoma, not known at the time of ATAAD. In the NOAC group, 1 patient died 6 months following redo surgery of redissection of the aortic root; another patient died after reoperation for a graft infection 12 months after the initial surgery. No additional deaths were reported in either group after 2 years.

Despite early diverging curves, survival analysis showed no significant difference between ATAAD operated on under NOAC and Coumadin (*P* = 0.059).

#### Coumadin/new oral anticoagulants versus no anticoagulation

**Figure 2:**
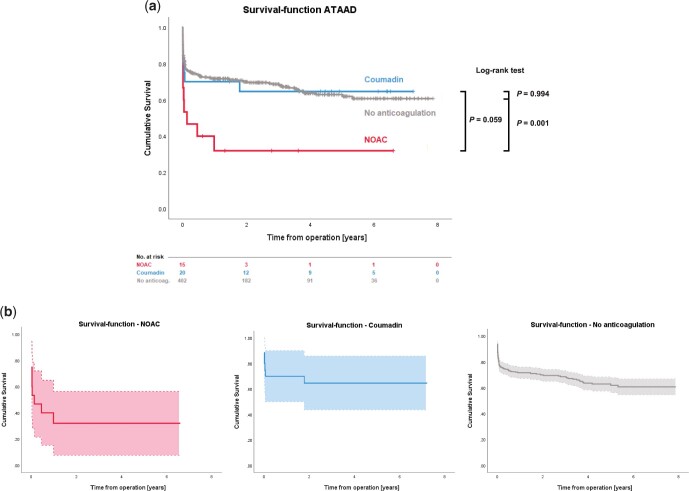
(**a**) Survival in patients treated for acute type A aortic dissection taking Coumadin, new oral anticoagulants and no anticoagulation. Coumadin versus new oral anticoagulants: *P* = 0.059; Coumadin versus no anticoagulation: *P* = 0.99; new oral anticoagulants versus no anticoagulation: *P* = 0.001. (**b**) Survival functions with 95% confidence intervals. Mean survival time [years]: new oral anticoagulants 2.2 ± 0.8; Coumadin 4.7 ± 0.8; no anticoagulation 5.2 ± 0.2.

In relation to the survival data of the 402 patients treated for ATAAD without prior OAC during the study period, survival rates were significantly lower for the NOAC group but not different for the Coumadin group (*P* = 0.001 vs *P* = 0.99). Kaplan–Meier survival plots for both groups as well as for the 402 patients without prior anticoagulation are depicted in Figs. 2a and b.

## DISCUSSION

In this retrospective study of 437 patients operated on for ATAAD, 35 (8%) had ongoing OAC. The use of NOAC in comparison to Coumadin was associated with higher morbidity, higher need for blood product transfusions and pronounced haemodynamic compromise. Survival analysis did not show a significant difference between patients taking NOAC compared to those taking Coumadin. When compared to patients who were treated for ATAAD without anticoagulation during the study period, the intake of NOAC was associated with significantly lower survival.

The use of NOAC for various cardiovascular conditions has increased, and short-term outcomes seem to support their safety. However, little is known about the incidence and management of late postoperative bleeding events. Only a few publications discuss management strategies for patients taking NOAC therapy in the presence of an emergency indication for cardiac surgery.

The largest study comparing complications and outcomes of anticoagulated patients treated for acute aortic disease (8 patients taking Coumadin vs 6 taking NOAC) was published recently [13] and reported that NOAC therapy was an independent risk factor for operative mortality. We confirm most of the findings of Bjornstad *et al.* [13] with a larger data set.

Our analysis shows a noticeably high operative mortality in the NOAC group (53%) compared to large registries such as the Canadian Thoracic Aortic Collaborative [14] (17.8%), the German Registry for Acute Aortic Dissection Type A (GERAADA) [15] (16.9%), the International Registry of Acute Aortic Dissection (IRAD) [16] (23.9%) as well as patients undergoing surgery for ATAAD in our own institution without prior OAC (24.4%).

This finding could be explained by the difficulties in managing haemostasis in patients on NOAC undergoing emergency aortic surgery. Although transfusions of blood products and coagulation factors may provide control over a pronounced bleeding situation in patients on Coumadin, bleeding management under NOAC therapy seems to be far more difficult.

The mean age of patients in our study was slightly higher than those in the 3 mentioned registries. Patients had more comorbidities, which resulted in a higher EuroSCORE II. Our in-hospital mortality in both OAC groups was comparable to that observed in the Bjornstad *et al.* study (67% NOAC vs 25% Coumadin) [13].

Risk calculation in ATAAD is challenging due to the variability of the presentation of the disease. Two IRAD risk models were introduced in 2007 [16] when NOAC were not yet available. One of the key findings with IRAD was that, regardless of the type of surgical procedure, patients presenting with either haemodynamic compromise or organ malperfusion are at almost twice the risk of perioperative death as patients who are stable at admission (31.4% vs 16.7%) [17]. Similar findings were observed in the GERAADA registry. In our series, both stable and unstable patients were equally distributed; expectedly, no difference was found in the risk calculations for either anticoagulated group.

As far as the IRAD risk model I is concerned, the predicted operative mortality is based solely on preoperative findings. In our study, the estimated risk was almost the same in both the NOAC and the Coumadin groups (24.4% vs 23.5%, *P* = 0.83). In the IRAD risk model II, which includes intraoperative findings, predicted operative mortality was found to be higher (although not significantly) in the NOAC group (37.6% vs 23.0%, *P* = 0.12). One explanation can be found in the pronounced bleeding complications noted in these patients. The main difference between both IRAD risk models is determined by intraoperative findings such as hypotension, right ventricular dysfunction or the need for concomitant coronary bypass grafting. Some of those findings are frequently observed when blood loss occurs, leading to massive transfusions, volume shifts, distributive shock and volume overload of the right ventricle.

The GERAADA score [18] that was introduced in 2020 is easy to calculate and certainly a useful tool to estimate the perioperative risk in ATAAD. Still, looking at the outcomes of the patients under NOAC in this analysis, the GERAADA score substantially underestimates the operative mortality risk by not taking into account the preoperative use of OAC.

Emergency surgical repair is the recognized treatment strategy in most patients presenting with an ATAAD. Fast diagnosis, aggressive blood pressure control and a stable transfer to a specialized centre are key factors for survival.

No major study has analysed whether a short delay until surgery to optimize preoperative risk factors and complete diagnostics may be favourable for certain patients (e.g. those with suspicion of coronary artery disease). Likewise, it is unknown if ongoing OAC may contribute to an increased incidence of bleeding or aortic rupture in the interval between diagnosis and surgery or if a short delay until surgery may be justified to improve drug-induced coagulopathy. A recent review by Sabe *et al.* [19] indicates that improvements in risk stratification should guide appropriate delay or permanent deferral of surgery in select individuals.

Haemadsorption (CytoSorb) has proven to be effective in accelerating the elimination of the platelet inhibitor ticagrelor and the NOAC rivaroxaban [20]. In the present study, surgery was delayed in 3 haemodynamically stable patients. The rivaroxaban-specific AFXa activity decreased from 203.7 µg/l to an average of 33.5 µg/l; only 1 patient had delayed sternal closure, and no in-hospital mortality occurred in this small subgroup. However, there is still no solid evidence to support a routine use of haemadsorption in the dissection scenario.

Even though 2 NOAC antidotes have recently received approval for sale in the United States and Europe, little is known about their use in the setting of ATAAD. The only publication found on PubMed.gov about the use of idarucizumab in patients with ATAAD unfortunately does not report patient outcomes or problems related to drug administration [21]. Besides, the high costs of around $25,000–$50,000 per patient for andexanet alfa (Ondexxya) and $3,000 for idarucizumab (Praxbind) still pose a major limitation for the routine use of these antidotes.

The presented data will maintain the debate concerning whether or not surgery for ATAAD in exceptional circumstances may be delayed and if this delay may improve operative outcomes [22]. Specifically for patients at highest bleeding risk, the question of having optimal framework conditions seems crucial. Although the situation with our 3 cases, i. e. their operations were delayed, may be a matter of debate, it has already been considered as an option by others [19, 23].

## LIMITATIONS

This is a single-centre retrospective observational study with a limited sample size. Despite equal distribution of all stated preoperative characteristics in a univariate analysis, group heterogeneity within reasonable limits may confound the conclusions made in this study.

Due to very localized dissection of the ascending aorta only, and the ability to exclude the whole dissection without circulatory arrest, 2 out of 20 patients in the Coumadin group and 1 out of 15 patients in the NOAC group were operated on under normothermia. Hypothermia is per se associated with pronounced coagulopathy. Although well distributed throughout both groups (10% vs 7%), these patients contribute to group heterogeneity and therefore may affect the study outcomes.

The survival analysis for patients without prior anticoagulation is not part of the study’s primary end point. Data for this group were not collected to the extent they were collected in the OAC group. Hence, when it comes to patient characteristics or treatment strategies, group heterogeneity cannot be ruled out, and intergroup comparisons in this case need to be interpreted with caution.

Due to the wide variability of ATAAD, different degrees of surgical repair and different strategies in peri- and postoperative care were required. Furthermore, several surgeons and anaesthesiologists were involved and may have contributed to different treatment concepts over the years.

Although standard operating procedures for managing blood products have been developed and followed as strictly as possible, minor adaptations to protocols have been made during the study period.

The indications and dosages of OAC were not under the control of the authors. Due to these limitations, there may be eventual bias concerning indications for specific therapies like open-chest treatment and transfusion of blood products.

## CONCLUSIONS

Emergency repair of ATAAD in patients with prior NOAC is associated with high morbidity and operative mortality.

Compared to Coumadin, NOAC therapy correlates with longer operating times, higher demands for transfusions of blood product and pronounced haemodynamic compromise. Survival does not differ between the 2 groups (*P* = 0.059), but, in contrast to Coumadin, patients on NOAC have significantly worse survival compared to patients who are operated on without any anticoagulants.

We emphasize that NOAC should be considered an independent risk factor for a bad outcome in emergency repair of ATAAD. New treatment strategies to further improve obtained results are required.

## DISCLOSURES

Carlos A. Mestres discloses consulting fees from the Edwards Clinical Events Committee (CEC) and Cytosorbents Corp. The other authors have nothing to disclose.

## DATA AVAILABILITY STATEMENT

All relevant data are within the manuscript and its supporting information files.

## FUNDING

No funding was provided for this publication.
